# A Reply to Dr. Allen’s Protest

**Published:** 1890-06

**Authors:** P. P. Wells

**Affiliations:** Brooklyn


### A REPLY TO DR. ALLEN'S "PROTEST."

DEAR HOMŒOPATHIC PHYSICIAN :—Though I have regarded myself as retired from the labors and responsibilities of writing for publication, by reason of age and its attendant infirmities, the "Protest" in your number for May of the current year seems to call on me to correct some errors therein, as my name is connected with the complaints of the protestant. He

* * * "feels compelled to write, complaining of the unfair treatment your journal pursues toward me [him] personally."

Though you are abundantly able to answer for yourself, and need help from no one, perhaps I may be permitted to say, without making myself chargeable with meddling with other persons' business, that I have been a constant and interested reader of your journal from its beginning, and though I have seen in it occasional allusions to the utterances of this protestant, not approving of them, I do not recall one which has seemed to me "unfair." These being as alleged, and I have seen no denial of this, the judgments of H. P. upon them have seemed to me *just* and *deserved.* In this case the only cure for the protestant's grief would seem to lie in his more careful utterances.

And then he charges that in my answer to the question: "What constitutes a Homœopathic Physician?" that

"Dr. P. P. Wells has seen fit in the last number of your journal to refer to *me* as ignorant."

This is a mistake. I did not refer to him at all as ignorant or otherwise. He was not in my thoughts when that paper was written. Indeed I did not know that he had been guilty of so great and silly an absurdity, as the answer given to the commissioner, as my authority, a common newspaper, said, it was given by quite another man. When told by a highly valued friend that this protestant had given this same definition, and that he (my friend) heard him, it was the first intimation I had had that there could be found *two* in any society so afflicted by strabismus as to see it possessed of so great power as merely by its membership to convert a mere man to a homœopathic physician. But so it is, and now this protestant says:

"Concerning my definition of a homœopathic physician, it is unassailable."

This may be so. We only know that it has been very generally laughed at. We know no one who has thought it worthy of other attention. But lest the outside world might think there was no better answer to this question of the commissioner, I attempted, not to "assail" this of the two members, but to give a definition of such physician, which seemed equal to the needs of all times and occasions.

So far are we from thinking this protestant "ignorant," we have said more than once, when those utterances of the H. P. which have called out the present complaint, have been spoken of—"*He knows better than he talks.*" We hope we did him no injustice in this judgment. We intended none, and should be truly sorry if compelled to believe we did any.

But the protestant, before this "unassailable" answer to the commissioner's question—"What do you consider *constitutes* a homœopathic physician?" gets things a little in a jumble, and in his protest, in endeavoring to make things clear, he tells, with all his might, what his society *calls* such a physician, which only answers a question no one has asked, and is of little interest to any outside his "society." The commissioner's question was, what *is?* The "unassailable" answer is what "our society" *calls* such—not exactly responsive to the question. The protestant does not appear to see the difference. But the readers of the protest, it may be supposed, will pardon something to a strabismus which sees only from *one* eye.

Then he is earnest in his endeavor to create belief in the orthodoxy of his teaching of true homœopathic therapeutics. This may be all as he says. But then how great must be his chagrin when so small evidence of such teaching is seen in the conversation and practice of the graduates sent out! One can only say before this saddening display—What a pity so great efforts should be followed by so small results!

Then, in his last paragraph he speaks rashly, and thus:

> * * * "Whatever may be said to the contrary, it is absolutely *true* that *every* physician in large practice is obliged to use other than homœopathic methods in the treatment of the sick, certainly not for their cure, but sometimes for their palliation." * * * [*Italics ours.*]

This shows how one may be led astray by his imagination, especially when this is interested in finding an excuse for one's wrong-doing. So far is this statement from being "absolutely true," we have no hesitation in declaring it absolutely *false*, and this from our own positive knowledge. In this we do not overlook the qualification, "*in a large practice*." This is quite a door of escape for one making rash assertions. Before one can be fastened in the falsehood he will have a definition of a "*large practice*," and this, where there is so gross strabismus as prevents distinction between one's imagination and facts, as in this case, may be difficult.

To meet this in the beginning we will say that in a practice of near half a century, which much of that time was as "large" as a tolerably active brain and fast horses made possible, there was in no one instance a resort to "other than homœopathic methods." A strict loyalty to law and obedience to its demands made a resort to means outside the demands of law wholly unnecessary. The need of such means was never felt. When the practitioner abandoned the practice and means of the school in which he had been somewhat carefully educated, for the practice and means of Homœopathy, it was because he had seen these last to be better—in these years he had never seen them otherwise—always better; and, properly administered, leaving no place for the abomination of palliation.

The whole amount then of this "absolutely true" declaration of this protestant, which is not true at all, is, that this is an open confession of what *he* does, and therefore he cannot see why others do not the same. It is only another display of that ever-afflicting strabismus, which prevents his distinction of the difference between truth and a gross slander of his colleagues.

BROOKLYN, May, 1890. P. P. WELLS, M. D.

